# Development and execution of a pandemic preparedness plan: Therapeutic medical physics and radiation dosimetry during the COVID‐19 crisis

**DOI:** 10.1002/acm2.12971

**Published:** 2020-07-11

**Authors:** Adam C. Riegel, Henry Chou, Jameson Baker, Jeffrey Antone, Louis Potters, Yijian Cao

**Affiliations:** ^1^ Department of Radiation Medicine Northwell Health Lake Success NY USA; ^2^ Zucker School of Medicine at Hofstra/Northwell Hempstead NY USA

**Keywords:** COVID‐19, pandemic, radiotherapy, SARS‐CoV‐2, treatment workflow

## Abstract

The SARS‐CoV‐2 coronavirus pandemic has spread around the world including the United States. New York State has been hardest hit by the virus with over 380 000 citizens with confirmed COVID‐19, the illness associated with the SARS‐CoV‐2 virus. At our institution, the medical physics and dosimetry group developed a pandemic preparedness plan to ensure continued operation of our service. Actions taken included launching remote access to clinical systems for all dosimetrists and physicists, establishing lines of communication among staff members, and altering coverage schedules to limit on‐site presence and decrease risk of infection. The preparedness plan was activated March 23, 2020, and data were collected on treatment planning and chart checking efficiency for 6 weeks. External beam patient load decreased by 25% during the COVID‐19 crisis, and special procedures were almost entirely eliminated excepting urgent stereotactic radiosurgery or brachytherapy. Efficiency of treatment planning and chart checking was slightly better than a comparable 6‐week interval in 2019. This is most likely due to decreased patient load: Fewer plans to generate and more physicists available for checking without special procedure coverage. Physicists and dosimetrists completed a survey about their experience during the crisis and responded positively about the preparedness plan and their altered work arrangements, though technical problems and connectivity issues made the transition to remote work difficult. Overall, the medical physics and dosimetry group successfully maintained high‐quality, efficient care while minimizing risk to the staff by minimizing on‐site presence. Currently, the number of COVID‐19 cases in our area is decreasing, but the preparedness plan has demonstrated efficacy, and we will be ready to activate the plan should COVID‐19 return or an unknown virus manifest in the future.

## 1 INTRODUCTION

The novel SARS‐CoV‐2 coronavirus was first detected in Wuhan, China, in December 2019. The first case of COVID‐19, the disease associated with the SARS‐CoV‐2 coronavirus, was diagnosed in the United States on January 20, 2020. The disease is primarily spread through respiratory droplets and close contact.^1^ Symptoms of COVID‐19 included fever, cough, shortness of breath, fatigue, muscle or body aches, headache, loss of taste or smell, sore throat, congestion or runny nose, nausea or vomiting, and diarrhea.^2^ Individuals with existing comorbidities such as hypertension, diabetes, and obesity are at increased risk for severe complications.^3^ Many people infected with the SARS‐CoV‐2 virus, however, remain asymptomatic^4^ and could unwittingly transmit the virus to others. Since the first diagnosis in January, the virus has spread to every state, infected over 1.9 million people in the United States, and claimed the lives of over 110 000 Americans as of this writing. Approximately 20% of the infections are in New York State and over half of these are in the five boroughs of New York City (Manhattan, Queens, Brooklyn, Staten Island, and the Bronx).

In addition to the acute effects of the virus itself, COVID‐19 has impacted all aspects of medical care including oncological care. Two studies from China found that cancer patients are more susceptible to contracting the virus.^5^, ^6^ One of these found that cancer patients experience worse outcomes than patients not undergoing cancer treatment.^6^ Radiation oncology departments present a particular challenge for restricting the spread of infectious disease due to daily treatments, full waiting rooms, and common equipment used by multiple patients such as the linear accelerator treatment couch.^7^ Recent publications from China,^1^ Singapore,^8^ and Italy^9^ have relayed some of the challenges associated with treating cancer patients during the COVID‐19 pandemic, but few have discussed the specific issues regarding medical physics support.

Northwell Health is a large health system with hospitals and clinics spread throughout the greater New York area, including the five boroughs of New York City, Long Island, and Westchester county. Northwell has treated thousands of COVID‐19 patients in the epicenter of the outbreak in the United States and recently published observations and outcomes for approximately 5700 patients.^3^ The Department of Radiation Medicine is localized to several key hospitals and outpatient centers in a variety of geographic locations in the health system. The administration and medical faculty acted quickly to develop contingency plans to ensure the continued safe operation of our department should an outbreak occur. The medical physics and dosimetry groups were tasked with developing pandemic operating procedures to ensure consistent quality of treatment while preserving staff safety and health. As the virus spread through downstate New York in early March, pandemic contingency plans were activated in our department. We continued to treat our patients, some of which were COVID‐19 positive.

The purpose of this manuscript is to describe the physics and dosimetry pandemic preparedness plan at our institution and assess its efficacy during the 2020 COVID‐19 pandemic. The benefits of this retrospective analysis are threefold: First, to share information from an early “hot spot” of the epidemic with our colleagues should they need to prepare; second, to consider our *ad hoc* readiness policies and procedures for more permanent adoption should COVID‐19 (or another pandemic) strike again; and third, to reflect on the potential evolution of large, multisite medical physics and dosimetry work as glimpsed during an extraordinary worldwide event.

## 2 MATERIALS AND METHODS

The first case of COVID‐19 in New York was diagnosed March 3, 2020 in New Rochelle, a small city in Westchester county close to New York City. It was at this time that the department began preparing in earnest for significant disruption of normal clinical activities.

The departmental administration identified five priorities to consider when developing contingency plans for said disruption: (a) Actively manage staff, (b) decrease treatment volume, (c) implement telehealth, (d) encourage multidisciplinary discussion, and (e) maintain a culture of safety.^10^

Medical faculty made several significant changes to reflect these priorities and reduce the potential for hospitalization.^11^ Reduction of patient volume was accomplished by prioritizing care into three categories: Priority I, II, and III. As described by Chen et al.,^11^ “Priority I” cases required radiation therapy most urgently, where loss of life, progression of disease, or permanent loss of function was possible. Examples included oncologic emergencies or advanced disease. “Priority II” cases could be delayed 4 weeks where the delay was unlikely to significantly impact patient prognosis. Examples included stage lung cancer, lymphoma, or benign brain conditions. “Priority III” cases could be delayed for 30 days or more where the delay was unlikely to impact patient prognosis. Examples included early stage breast or prostate cancer. Prioritization was decided by the attending radiation oncologist and presented at daily contouring rounds. Additional changes included preferentially choosing hypofractionated treatment regimens if clinically reasonable, spacing out treatments to reduce crowding in waiting rooms and in hallways, disinfecting all common surfaces between patients, conversion of all meetings to video conference, and increasing communication with staff to transparently share updated information when available. The medical physics and dosimetry group adapted these general guidelines for our specific clinical contributions. Strategies are shared in subsequent sections.

### 2.A. Staff management and remote work

Our first task was to identify what physics and dosimetry activities could be performed remotely to most effectively enact social distancing. Essential clinical physics responsibilities were split roughly into three categories: External beam treatment planning, special procedures, and hardware quality assurance (QA).

External beam treatment planning (which is also the primary domain of the dosimetry group) included image registration, normal tissue contouring, treatment planning, and all reviews/approvals associated with the planning process. Our department utilizes Velocity v.4.0 (Varian Medical Systems, Atlanta, GA) for the majority of image registration and contouring, Eclipse v.15.4 (Varian Medical Systems, Palo Alto, CA) for treatment planning, and Mosaiq v2.64 (Elekta, Sunnyvale, CA) for record and verify. Several years ago, we developed a web‐based electronic “whiteboard” to track completion of treatment planning tasks and facilitate automated handoffs between care team members.^12^

Fortunately, due to the geographically dispersed nature of our multisite department, most of these systems were already remotely accessible. Velocity, Mosaiq, and the whiteboard were accessed through the health system virtual private network (VPN), and Eclipse was accessible securely via Citrix on an externally managed cloud server. Additionally, many meetings, including daily contouring rounds, research meetings, faculty meetings, and staff meetings, were already being held remotely in order to include team members from all health system sites. The biggest hurdle was ensuring that all physicists and dosimetrists had functional access to these systems from home or offsite locations. Most staff members were utilizing private computers for remote access which made uniform installation and troubleshooting difficult. Physicists and dosimetrists were asked to check their remote access and update with our departmental informatics group if necessary.

At our institution, special procedures included high‐ and low‐dose rate brachytherapy, total body irradiation, GammaKnife stereotactic radiosurgery (SRS), linac‐based radiosurgery and stereotactic body radiation therapy (SBRT), and a variety of interventional procedures. Each special procedure required a dedicated physicist for coverage. The reduction of patient volume by medical faculty, however, drastically cut the number of procedures in our department. With the exception of urgent GammaKnife SRS, SBRT, and limited high‐dose rate brachytherapy, all other procedures were postponed. This significantly reduced the need for on‐site coverage by physics.

Quality assurance over this particular time period included patient‐specific QA, monthly QA, and clinical troubleshooting. Patient‐specific QA is always performed after hours or on the weekend and thus was relatively unaffected as QA staff were not placed at significant additional risk. Similarly, monthly QA was planned such that contact with other patients and staff members was minimized. Non‐urgent machine service was consolidated to limit vendor visits to the clinic.

Under normal circumstances, there is an on‐call physicist at each radiation medicine site in the health system. At our largest clinical site, there is a morning on‐call physicist and an evening on‐call physicist. For the COVID preparedness plan, we eliminated all on‐site on‐call physicist duties except the morning and evening on‐call physicists at our main clinical site. These physicists would act as on‐call for the entire health system and, if local troubleshooting was required, the on‐call physicist would contact nearby physicists who could travel to the clinic, perform the necessary maintenance, and return home. Similarly, dosimetrists provided on‐site coverage with one dosimetrist at our main clinical site during treatment hours.

All physicists and dosimetrists who were not needed for on‐site coverage or procedures were asked to work remotely. Physicists and dosimetrists who were present on‐site were provided protective equipment including surgical masks, gloves, and ample disinfectant for routine wipe downs of workstations and equipment.

### 2.B. Communication

For physicians, multidisciplinary cooperation meant cooperation between physicians of varying specialty, particularly medical oncology and surgical oncology. For physicists and dosimetrists working remotely, this meant decreasing verbal and written communication barriers with other radiation oncology team members. This was accomplished several ways. Email and phone (both voice and text) remained the primary means of communication. Email was supplemented, though, with other communications platforms like Microsoft Teams which provided both text communication, file sharing, and teleconferencing for more complex discussions. With in‐person meetings temporarily suspended, Microsoft Teams was used for clinical discussions and Zoom was used for teaching medical physics graduate classes. All communication software tools used in clinical activities were encrypted to HIPAA‐compliant standards.

### 2.C. Analysis of efficiency and quality during the pandemic

It was extremely important to maintain high‐quality standards for our patients. Previously, we have reported the development and use of our checklist‐based “No Fly” system.^13^, ^14^ It was emphasized to all staff that the “No Fly” system should be followed and safety should not be compromised due to the added complexity of the pandemic.

The efficacy of our preparedness plan was analyzed in several ways. First, we calculated the number of work hours (defined between 8:00 AM and 6:00 PM) elapsed between handoffs in the external beam treatment planning process. Second, we assessed the timeliness of first day chart checks, weekly chart checks, and final chart checks. Third, we reviewed the radiation oncology incident learning system (ROILS) entries to assess the impact on safety. Each metric was compared to a similar time frame the prior spring (March 22, 2019 to May 6, 2019). Fourth, we issued a confidential web‐based survey to all physicists and dosimetrists to gauge their opinion of the altered work arrangements during the COVID crisis.

## 3 RESULTS

Readiness planning for the pandemic began on March 9, 2020. The plan was activated March 23, 2020 and data were collected until May 6, 2020. The department consists of 20 physicists, 14 dosimetrists, and 8 quality assurance technicians. During this 6‐week time period, one to two dosimetrists were present on‐site each work day with the remainder working remotely. Based on the remaining scheduled procedures and assigned on‐call, we estimate that six to seven physicists were on‐site during treatment hours on any given day with the remainder working from home. One dosimetrist contracted COVID‐19 and was quarantined for 3 weeks. One dosimetrist had symptoms consistent with COVID‐19 and was quarantined for 1 week. Six physicists were quarantined for 2–3 weeks each due to symptoms consistent with COVID‐19, prolonged exposure to someone with confirmed COVID‐19, or travel to an area where an outbreak occurred. From what we can ascertain, however, it appears that quarantine efforts were fruitful in that the virus did not spread among team members. If team members were symptomatic, they were not assigned work until they recovered. If team members were quarantined but asymptomatic, they were asked to perform clinical duties remotely.

A total of 263 patients were planned during this time period. Two‐hundred and seven were planned on the “standard” timeline (longer timeline for conformal and intensity‐modulated plans) and 56 were planned on the “urgent” timeline (cord compressions, bleeding, various palliative treatments, etc.). This compares to 354 in 2019 with 296 on the standard timeline and 58 on the urgent timeline. This represents a 30% drop in standard timeline cases and an overall reduction in patient volume of 25%. For comparison, the total dosimetrist FTE increased by 2 from 2019 to 2020. The total physicist FTE decreased by 1 in the same time period.

**Fig. 1:**
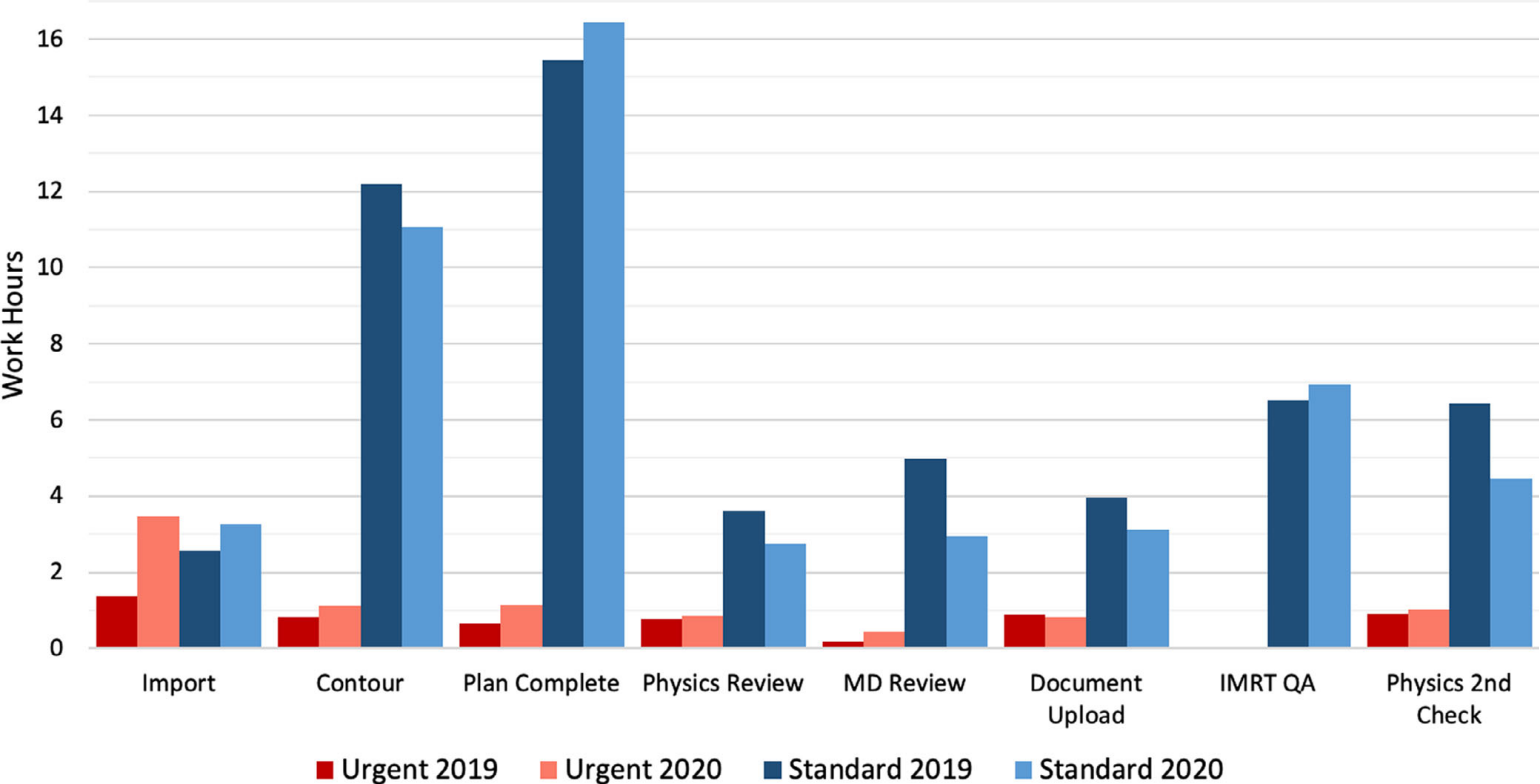
Planning efficiency during COVID‐19 altered work arrangement. “Urgent” timeline includes cord compressions, palliative treatments, etc. “Standard” timeline includes three‐dimensional‐conformal and intensity‐modulated plans. Data were collected over comparable six‐week time period in 2019 and 2020.

**Fig. 2:**
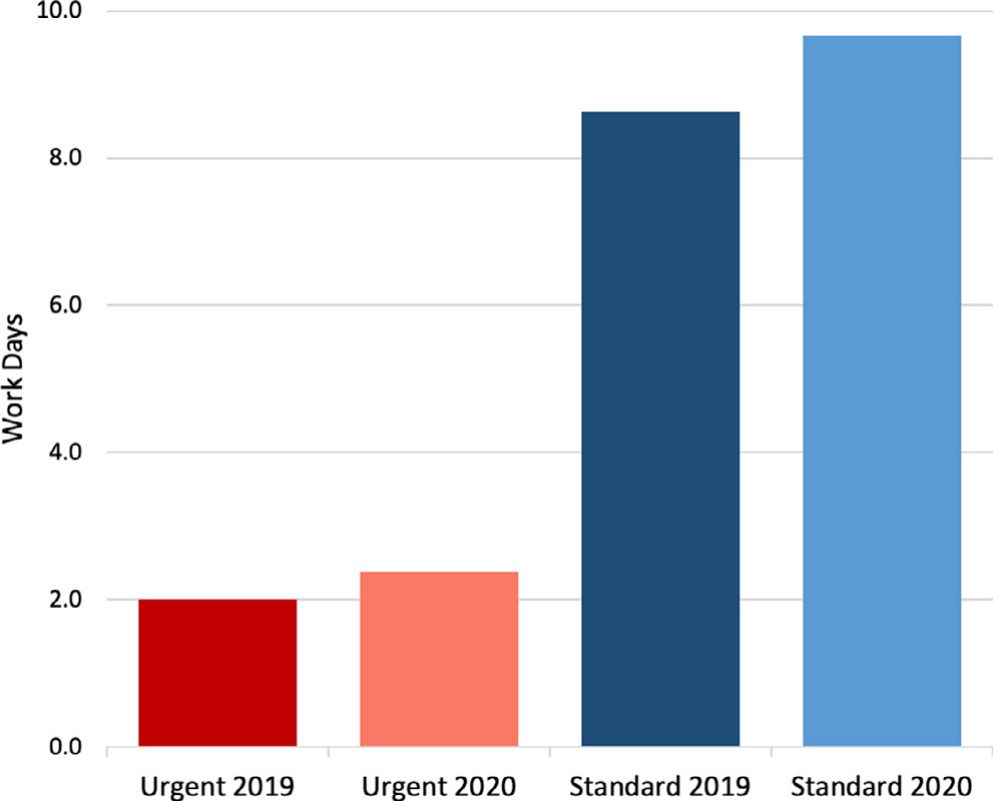
Time in workdays from computed tomography simulation to virtual simulation (dry run) on linear accelerator for urgent and standard timeline cases.

Analysis of treatment planning efficiency is shown in Fig. 1. Several steps of the process were completed more quickly for the standard timeline with the majority of staff working remotely, including contouring, initial physics review, initial physician review, plan documentation upload, and second check. Urgent cases showed modest increases in completion time. With the exception of image import, median values increased by <1 hour. The total number of workdays between CT simulation and virtual simulation (dry run) on the linear accelerator are shown in Fig. 2. The time increased by slightly over 1 day for standard treatments (8.6 vs 9.7 workdays) and just under half a day for urgent treatments (2.0 vs 2.4 workdays) during the COVID crisis.

The number of first day, weekly, and final physics chart checks decreased in 2020 compared to 2019. First day checks decreased by 37% (283 in 2019 vs 177 in 2020). Weekly and final physics chart checks decreased by 22% (701 vs 549 weekly, 245 vs 192 final physics checks). The first day checks likely decreased more due to delayed treatment starts. Weekly and final physics checks were less impacted due to continuing patients who had already started treatment.

Chart check efficiency yielded similar results to treatment planning. First day chart checks were completed in 0.4 median workdays in 2019 (range: 0–6.5 days) and 0.4 median workdays in 2020 (range: 0–5.5 days). Weekly chart checks were completed in 5.2 median workdays in 2019 (range: 0.2–15.4 days) and 4.9 median workdays in 2020 (range: 0.2–14.5 days). Final physics checks were completed in 0.7 median workdays in 2019 (range: 0–9.9 days) and 0.5 median workdays in 2020 (range: 0–11.3 days).

The number of physics‐related events in our local ROILS database decreased by 68% in 2020 when compared to the same time frame last year. Part of this decease, however, can be attributed to a 50% drop in the total number of reported incidents, most likely due to a natural deprioritization of reporting during the COVID‐19 event.

The web‐based survey was completed by 31 of 42 physics/dosimetry group members. Although 76% of respondents reported having all the tools they needed to perform their work remotely, 39% said that configuring remote access was somewhat to very difficult and 63% reported connection problems at least once per week, with 20% reporting connection problems multiple times per day. Despite this, 83% stated their transition to remote work was “neutral,” “somewhat easy,” or “very easy.” Large majorities reported motivation and focus equivalent to or better than that experienced in the office (87% and 83%, respectively), with 71% estimating they were just as or more efficient working remotely compared to the office. This is despite more than half of respondents (54%) reporting additional responsibilities beyond work, for example, caring for children (38%) or caring for sick family members (10%). Nearly half still found email as the best way to communicate (48%) with text messaging close behind (26%). Participants were also asked, given their experience during the COVID‐19 crisis, how they would prefer to work in the future. The results were mixed: 13% preferred entirely in‐office, 13% preferred entirely remote, 29% preferred mostly office with 1–2 days remote, 22% preferred mostly remote with 1–2 days in‐office, and 22% preferred an even split between office and remote work.

Ten of 31 participants left free form written responses about their work experience during the COVID‐19 crisis. In general, people were satisfied with the current arrangements and spoke favorably about working remotely. Some expressed relief in limiting their exposure to the virus, others spoke about being more focused and efficient at home. Several participants mentioned they missed social interaction with their colleagues on a daily basis.

## 4 DISCUSSION

The impact of COVID‐19 on clinical operations in a radiation oncology department cannot be overstated. In the past 2 months, numerous publications have provided recommendations for radiation oncology clinics to remain operational during the most significant public health crisis in a generation. Zaorsky *et al*. recommend the “RADS” framework, which stands for “Remote visits, Avoid radiation, Defer radiation, Shorten radiation.”^15^ Several site‐specific recommendations have been published to aid physicians in decisions of avoiding or deferring radiation and toward specific fractionation regimens to shorten radiation.^16^, ^17^, ^18^, ^19^ Many of these works, however, were accepted for publication and made available online after the coronavirus began to spread exponentially in the greater New York area. The purpose of the current work was to evaluate a pandemic preparedness plan in a therapeutic medical physics and dosimetry service in a hospital‐based radiation oncology clinic located in the epicenter of the United States COVID‐19 pandemic.

In retrospect, many components of our contingency plan reflect recently published recommendations. On March 24, the day after the majority of our staff began remote work, the AAPM published a letter authored by Dr. Brent Parker, chair of the AAPM Professional Council. This letter contained several important recommendations specifically for medical physicists, including development of alternative staffing models, backup physics coverage, prioritization of quality assurance, establishing adequate resources for remote work, and properly sanitizing all shared physics equipment.^20^ Our plan overlaps substantially with these and other suggestions in the letter.

In a letter to the editor, Li *et al*. make several recommendations for medical physicists based on their experience in Henan Cancer Hospital in Zhenghzhou, China.^21^ These recommendations focused primarily on infection control: Personal protective equipment protocols, office cleaning procedures including frequency and materials, temperature monitoring at access points, and social distancing. Although the authors mention dividing their workforce and adjusting the workload, there are few details about their efforts in this regard. It is possible the authors focused on maintaining standard staff levels while increasing disinfection frequency and personal protective equipment. At our institution, we took advantage of the remote infrastructure already in use for our geographically spread department to maximize social distancing and minimize the need for vigorous disinfection in physics and dosimetry office spaces in the department. One significant deviation between our infection control protocols and those in China: We did not monitor asymptomatic patient or staff temperature on a regular basis, rather relying on observable symptoms and contact history to determine risk of infection. Given the prevalence of asymptomatic COVID‐19 positive patients,^22^ we will consider integrating this check into our standard protocols.

One of the problems with the current scenario is that many of us have little experience in delivering care during a natural disaster. Based on the experience during Hurricane Maria in Puerto Rico, Gay et al. propose the “PCOC” paradigm: Prepare, Communicate, Operate, Compensate.^23^ Although lessons learned from a hurricane are not directly transferrable to a pandemic, the authors’ recommendations can certainly provide a framework for how to prepare a medical physics and dosimetry group for other events like the current pandemic. “Prepare,” for example, could be establishing and verifying remote connectivity for all team members, creating alternate staffing models to decrease exposure risk, ensuring an adequate supply of personal protective equipment for staff, or obtaining disinfectants that can be used safely on physics equipment with sensitive electronics or plastics. “Communicate” is exemplified by transparent communication from administration to the entire department and clear, precise communication to justifiably concerned cancer patients that their care will not suffer due to delays or other changes in the department. Fortunately, we have not experienced the existential “Operate” issues described by Gay *et al*. such as limited electricity, water, and food. “Operate,” however, could simply mean the faithful execution of the pandemic preparedness plan. “Compensate” as defined by the authors in the context of a hurricane is transferrable to the pandemic. Physicians are encouraged to alter treatment plans and fractionation schedules to ensure adequate dose is delivered to the patient if/when delays occur.

The results of the current study show that external beam planning operations were not substantially impacted by the pandemic preparedness plan (Fig. 1). Most steps of the treatment planning process were completed as quickly as or more quickly than a comparable 6‐week time period in 2019. The time between CT simulation and dry run, however, was slightly longer, most likely due to the prioritization of patients and forced delays in treatment start date (Fig. 2). First day, weekly, and final chart checks were also completed in 2020 as quickly as or more quickly than 2019. There are, however, two major caveats in these findings. First, due to prioritization and subsequent reductions in non‐urgent care, the total planning load was reduced by 25% in 2020 compared with the same 6‐week interval in 2019. The number of chart checks was similarly reduced by 22–37%. Second, special procedures, with the exception of a few urgent cases, were eliminated. These two factors mean that dosimetrists had fewer plans to generate and physicists, who are primarily responsible for procedure coverage, were more available for planning and chart checking, both of which most likely lead to faster turnaround times. One drawback to our study was that we did not measure response time for on‐site machine troubleshooting. With the exception of our main clinical site, physicists were called in from home to work on machines. Response times necessarily increased to include the physicist’s commute.

Overall, the transition from normal operations to limited, remote operations was smooth and clinical efficiency was relatively unaffected. We attribute the majority of this success to technological infrastructure, centralized treatment directives,^24^ and workplace culture established in our distributed, multisite environment over the past decade. In many ways, physics and dosimetry were already operating remotely: Dosimetrists at one site were planning for another site, physicists were checking plans from all over the system, team members were meeting via video conference, and physicists were rotating between sites on a regular basis to provide coverage for procedures and quality assurance. The electronic whiteboard was critical in keeping the treatment planning workflow organized, up‐to‐date, and accessible to anyone on the departmental network. Based on the promising results of the current analysis, we summarize our recommendations for pandemic preparedness planning in Table 1. We believe these recommendations are in‐line with those published by other sources and could be generalized to other natural disasters with sensible customization to the situation and local needs.

**Table 1:** Guidelines for medical physics and dosimetry operations during shelter‐in‐place conditions.

Reduce the number of people in the department at any given time to reduce the risk of disease transmission
Work with physicians to prioritize patients based on urgency of care. Cases that can be delayed without significant risk of disease progression or loss of function should be delayed
Reduce on‐site clinical coverage to skeleton crew
If on‐site troubleshooting is required, assigned physicists should address the problem on‐site while observing social distancing and donning proper protective equipment. Once the problem has been resolved, physicists should return home to work remotely
Utilize remote access to clinical software as much as possible
Prepare backup coverage in case physicists become infected or quarantined
Establish and maintain clear lines of secure communication between physics, dosimetry, and other team members

It is tempting to view the current work situation as an opportunity to “test‐drive” remote medical physics work and extrapolate to non‐emergent (i.e., non‐pandemic) conditions. Remote work by physics and dosimetry, even if utilized part time as described in our survey, could be advantageous. Physicists and dosimetrists could experience reduced commuting time, increased schedule flexibility, and a more focused environment with fewer interruptions. Employers could hire employees across the nation without requiring a physical presence on‐site. Administrators would need fewer physical offices and workspaces for physicists and dosimetrists, an advantage not limited to radiation oncology.^25^ Although the caveats listed in the previous paragraph cast doubt on the scalability of these findings to full patient load, a diverse and demanding array of special procedures, and longer term clinical projects such as acceptance or commissioning of new equipment, which are most likely on‐hold at the current time, our survey certainly indicates there is interest in working remotely. We will investigate the feasibility of integrating remote work into standard clinical practice while maintaining a robust physical presence.

## 5 CONCLUSION

The COVID‐19 crisis has profoundly impacted the United States healthcare system. Radiation oncology is particularly exposed to disruption due to the vulnerable nature of our patient population and the logistics involved with recurring therapy on shared equipment. This manuscript described the actions taken by our medical physics and dosimetry group to ensure high‐quality radiation therapy could be delivered safely and effectively to our patients in the midst of a pandemic. At the time of submission, the number of new cases in New York City and State is slowly decreasing. We are, however, preparing for a resurgence of the disease. Given our experience the past 6 weeks, we will refine and formalize our pandemic preparedness plan and will be ready to activate the plan should the need arise.

## CONFLICT OF INTEREST

The authors do not have any conflict of interest to disclose.
